# Variations in motility and biofilm formation of *Salmonella enterica* serovar Typhi

**DOI:** 10.1186/1757-4749-6-2

**Published:** 2014-02-05

**Authors:** Kalaivani Kalai Chelvam, Lay Ching Chai, Kwai Lin Thong

**Affiliations:** 1 Institute of Biological Sciences, Faculty of Science, University of Malaya, Kuala Lumpur, Malaysia; 2 Laboratory of Biomedical Science and Molecular Microbiology, Institute of Graduate Studies, University of Malaya, 50603 Kuala Lumpur, Malaysia

**Keywords:** *Salmonella* Typhi, Biofilm, Motility swarming, Swimming

## Abstract

**Background:**

*Salmonella enterica* serovar Typhi (*S*. Typhi) exhibits unique characteristics as an intracellular human pathogen. It causes both acute and chronic infection with various disease manifestations in the human host only. The principal factors underlying the unique lifestyle of motility and biofilm forming ability of *S*. Typhi remain largely unknown. The main objective of this study was to explore and investigate the motility and biofilm forming behaviour among *S*. Typhi strains of diverse background.

**Results:**

Swim and swarm motility tests were performed with 0.25% and 0.5% agar concentration, respectively; while biofilm formation was determined by growing the bacterial cultures for 48 hrs in 96-well microtitre plate. While all *S.* Typhi strains demonstrated swarming motility with smooth featureless morphology, 58 out of 60 strains demonstrated swimming motility with featureless or bull’s eye morphology. Interestingly, *S.* Typhi strains of blood-borne origin exhibited significantly higher swimming motility (P < 0.05) than stool-borne strains suggesting that swimming motility may play a role in the systemic invasion of *S.* Typhi in the human host. Also, stool-borne *S*. Typhi displayed a negative relationship between motility and biofilm forming behaviour, which was not observed in the blood-borne strains.

**Conclusion:**

In summary, both swimming and swarming motility are conserved among *S*. Typhi strains but there was variation for biofilm forming ability. There was no difference observed in this phenotype for *S*. Typhi strains from diverse background. These findings serve as caveats for future studies to understand the lifestyle and transmission of this pathogen.

## Background

Motility and biofilm forming capability in bacterial pathogens are one of the most studied bacterial physiology nowadays as these characteristics have important roles on pathogenicity [1-3]. Almost all of the identified pathogenic bacteria of humans, such as *Vibrio cholerae*, *Pseudomonas aeruginosa*, *Salmonella* and pathogenic *E. coli*, are motile. However, the ability to form biofilm is variable in human bacterial pathogens.

Typically, bacterial motility refers only to swimming motility in diagnostic microbiology. The typical bacterial motility test performed in the clinical laboratory based only on the ability of bacterial cells to migrate away from the semi-solid stab. In fact, bacteria move in various modes, including swimming and surface swarming. Swimming occurs when bacterial cells move in the aqueous environment (low agar concentration) while swarming motility is a collective behaviour of bacterial cells associated with migration on semi-solid surfaces [4]. Unlike the classical swimming motility in aqueous environment, vegetative cells must first differentiate into elongated and hyperflagellated swarmer cells to migrate on the surface [4,5]. Besides the obvious physical changes, swarmer differentiation can also be coupled to increase expression of important virulence determinants in some species. Besides that, swarming is also linked to biofilm forming ability in bacteria, which serve as another important virulent factor of human pathogens [6,7].

A biofilm is defined as bacterial colony adherence to solid surface that secretes a self-initiated, protective exopolysaccharide matrix [8,9]. The ability to form biofilms through the complex interaction of bacteria has been reported to be important for bacterial survival within the human host. Moreover, both the innate and adaptive immune responses of the human hosts might not be able to eliminate the pathogen within the well-established biofilm [10,11].

Motility and biofilm-forming ability have been reported in *Salmonella enterica* serovar Typhi (*S*. Typhi) [12]. It is the etiological agent of typhoid fever, infecting 21.7 million people and causing 217,000 deaths annually [13]. Several case-control studies have investigated risks for enteric fever; the majority implicate water and food as important transmission routes [14-17]. Most patients who recover from the infection are able to eliminate the bacterium completely from their bodies. However, an approximately 5-10% of infected individuals may remain as carriers, continuously shedding *S.* Typhi in their stools [18]. A recent study showed that *S.* Typhi is frequently associated with the presence of gallstones in asymptomatic human carriers, in which the pathogen colonises and persists as biofilm on the gallstones [12]. Despite the caustic nature of bile in gallbladder, biofilms allow the continual shedding and reattachment of individual cells, contributing to the spread of bacteria via urine and faeces, particularly in the human host [19,20].

Motility has also been detected in *S*. Typhi. Indeed, intact motility (swimming motility) has been identified to be an invasive-related factor of *S*. Typhi [21]. Among more than 2500 serovars of *Salmonella enterica*, *S*. Typhimurium is one of the earliest serovars being identified to undergo morphological differentiation into swarmer cells [5]. Later, Kim and Surette [22] continued to screen for the ability of surface swarming among different *Salmonella* serovars and have observed swarming motility in most of the strains studied, including *S*. Typhi. They concluded that swarming could represent an evolutionarily conserved behaviour in *Salmonella*. However, in their study, only two strains of *S*. Typhi were tested. Hence, in this study, we have extended Kim and Surette’s work to look into both swimming and swarming motility in more strains of *S*. Typhi of various origins to determine if variation in motility, specifically surface swarming exist in *S.* Typhi.

This work aims to provide an insight into the swimming and swarming motility and biofilm forming ability in *S.* Typhi. We have selected strains to represent various countries (Malaysia, Indonesia, Papua New Guinea and Chile); years (from 1983 to 2008); and different disease manifestation origins (strains from stool or blood samples of typhoid patients and asymptomatic human carriers) to demonstrate all possible variations within *S*. Typhi. The inclusion of *S*. Typhi strains from diverse background will provide us with a better and more truthful insight into the possible physiological variation in this strict human pathogen.

## Results and discussion

### *S.* Typhi strains demonstrated both swarming and swimming motility

**Figure 1:**
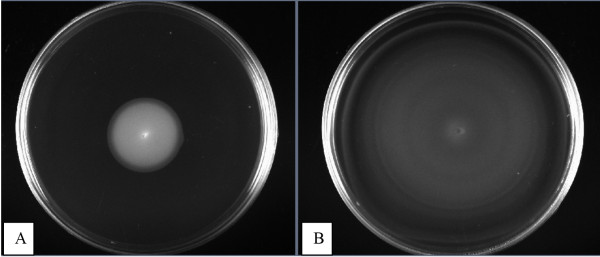
**Swarming and swimming behaviour of *Salmonella enterica *serovar Typhi.** Swarm medium is nutrient broth (0.5% agar) and swim medium (0.25% agar) supplemented with glucose as carbon source (0.5% [wt/vol]). Uncolonised agar is black and bacteria biomass is white. All images were captured after 24 hr at 37°C. Panel **A** shows swarming motility. Panel **B** shows swimming motility.

The motility of 60 strains of *S*. Typhi was measured for their surface swarming (growth in media with 0.5% agar) and swimming (growth in media with 0.25% agar) ability. All the strains were able to swarm across the agar surface and formed featureless, smooth and flat colony (Figure 1A). Featureless colonies are made when cells spread evenly and continuously outward from the point of inoculation, as a monolayer. The monolayer is transparent but may be seen when incident light is reflected off the surface or when oblique light is transmitted through the agar. Cell density in the monolayer is high and approximately uniform throughout the swarm, increasing slightly at the advancing edge [23]. When the monolayer reaches the boundary of the plate, the colony grows into a featureless mat [24].

However, the swarming motility of the strains tested in this study was weaker compared to the study of Kim and Surette [22].

In this study, all of the tested strains for swarm motility assay were measured more than 1.5 cm - 7.7 cm after 24 hrs of incubation. Strains showing migration of cells (increase in colony diameter) were considered as positive swarming. Initially when this work was done, the rates of the migration of bacteria from the point of inoculation were measured at 0, 6, 12, 18 and 24 hr. However in this study, at 0 hr, *S*. Typhi strains showed no migration. At 6 hr and 12 hr, *S*. Typhi strains showed similar rate of migration. After 18-24 hrs, most of the *S*. Typhi strains colonised the entire surface of the petri plate and reached the maximum size. Among the 60 strains tested, UJ308/98 demonstrated the most active swarming motility. Interestingly, this strain was isolated from blood specimen of a deceased typhoid patient in Papua New Guinea, in which typhoid fever is highly endemic. In this study, we had also included *S.* Enteritidis and *S*. Typhimurium as the control strains for motility tests. Our results showed that both *S*. Enteritidis and *S*. Typhimurium were able to swarm on the agar surface, with *S*. Enteritidis showing more active swarming motility than *S*. Typhimurium and *S*. Typhi. Both *S*. Typhimurium and *S*. Typhi had comparable swarming motility.

Most strains exhibited featureless swimming pattern on the agar media (93.4%) while one *S.* Typhi strain (ST02/08) showed bull’s eye (Figure 1B). Bull’s eye is a typical swim pattern which is also known as zones of consolidation terraces, caused by sequential rounds of swarm cell differentiation and swarming colony migration [6]. The most studied bull’s eye pattern is formed by *P. mirabilis* which differentiates into swarm cells that are multinucleate, 20-50 fold elongated and express thousands of flagella on a solid surface [25,26]. It is likely that counter-clockwise or clockwise switching patterns of the flagella motors influence the direction of cell movement and hence the patterns. However, the relationship of these patterns to the virulence mechanism has not been studied in these cases.

Although a majority of the strains were highly motile in less viscous media, 2 strains (ST319/87 and TP3/97) were not able to swim. We re-confirmed this result with the Sulfide-Indole-Motility (SIM) test tube assay and found the same outcome. In fact, motility is an important bacterial virulence factor that aids in the gut colonisation to initiate infection in human host. To confirm this finding, two motility-associated and flagellin related genes, *fliC* and *flgK*, were selected to test on all the strains. The flagellar filament of *S. enterica* is approximately 10 μm long and is comprised of two antigenically distinct flagellin proteins, FliC (H:*i*) and FljB (H:*1*,*2*). Previous experiment conducted by Crawford and co-workers [18] demonstrated that flagellar subunit *fliC* is critical for binding to cholesterol in serovar Typhimurium. On the other hand, *flgK* gene functions as a hook filament junction. In this work, these genes were studied to test for the presence of these genes and whether it contributes to the motility of *S*. Typhi. Both *fliC* and *flgK* genes were present in all the strains tested. However, there are other important genes for motility which were not studied in this work. Moreover, these 60 unique *S*. Typhi strains studied in this work were previously subtyped by pulsed-field gel electrophoresis (PFGE) and these strains were genetically different (unpublished data). The result suggests that the loss of swimming motility in these two strains (ST319/87 and TP3/97) could be due to the loss of other motility related genes or other factors which were not tested in this study.

**Figure 2:**
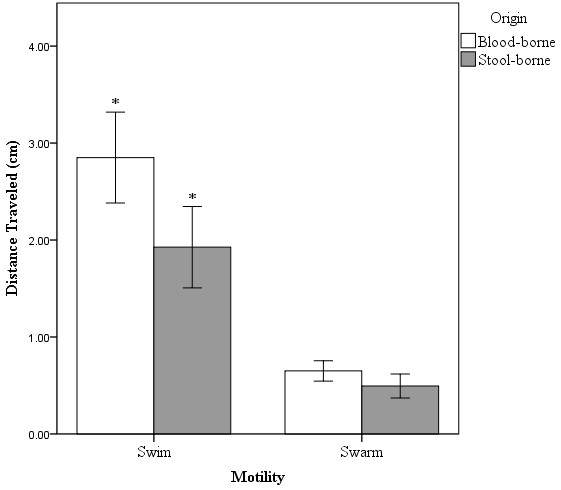
**The Motility of *Salmonella *Typhi in Blood-borne and Stool-Borne Strains.** This figure shows comparison between blood-borne, n =24 (white) and stool-borne, n =19 (gray) *S.* Typhi in swim and swarm motility assays. Y-axis indicates the distance traveled (cm) in motility assays. Vertical lines associated with histogram bars represent standard error of the mean. * implies *p* < 0.05. There was a significant difference observed only in swim motility assay with *p* < 0.05 for blood-borne strains compared to stool-borne *S*. Typhi strains. However, swarm motility showed non-significant differences in both blood-borne and stool-borne *S*. Typhi strains.

In this study, we found a significant difference in the swimming motility between blood-borne and stool-borne *S*. Typhi strains (P < 0.05) (Figure 2). *S*. Typhi strains isolated from the blood specimen of typhoid fever patients exhibited higher swimming motility level than the strains isolated from stool specimens. We did not observe any significant difference in the surface swarming between blood- and stool-borne strains (P > 0.05) (Figure 2). Interestingly, in our previous study on the carbon catabolism among *S*. Typhi strains [27], blood- and stool-borne strains differed in their carbon catabolic profiles. These findings suggested that blood-borne and stool-borne strains may represent two different pathogenesis stages, systemic invasion and persistence in the human host, respectively. While this finding suggests motility may play a role in the systemic invasion of *S*. Typhi in the human host, more in-depth study is needed to clarify this speculation. From a biological viewpoint, such differences in motility raise the possibility that strains with high motility may be more capable of swimming through the intestinal mucus and replicate within macrophages and infected phagocytes [28]. Replication of the bacteria within macrophages in the liver and spleen resulted in the release of the pathogen in the bloodstream which later invades the gallbladder [29]. *S*. Typhi is then adapted and persisted in the gallbladder, in which high motility is not necessary, and later leads to bacterial shedding in the urine and faeces.

### *S*. Typhi strains exhibited red, dry and rough (RDAR) morphotype

**Figure 3:**
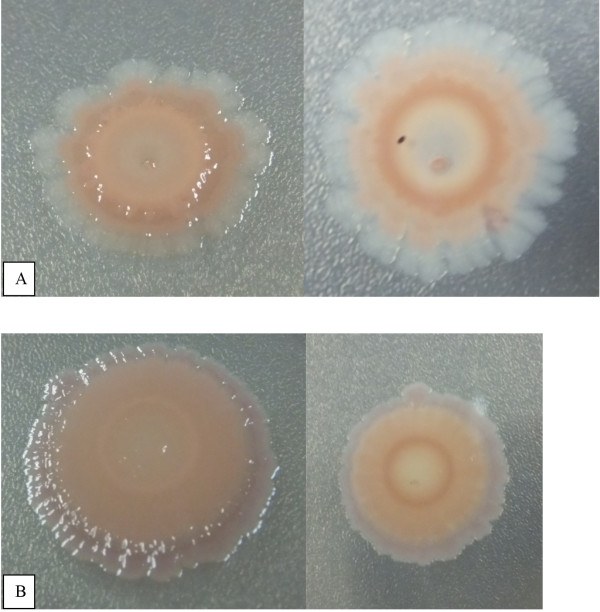
**RDAR Morphotype in *Salmonella *Typhi.** Morphotype of red, dry and rough (RDAR) colony which shows presence of curli and cellulose in *S.* Typhi and *S.* Typhimurium after 7 days of cultivation at 37°C on Congo Red Agar. Panel **A**: Morphotype of *S.* Typhi. Left panel and right panel are the front view and back view of *S*. Typhi RDAR colony morphotype on Congo Red Agar, respectively. Panel **B**: Morphotype of *S.* Typhimurium as a control. Left panel and right panel are the front view and back view of *S*. Typhimurium RDAR colony morphotype on Congo Red Agar, respectively.

The components of exopolysacharides (EPS) that have been identified in *Salmonella* spp. biofilms include cellulose, colanic acid, the Vi antigen, curli fimbriae, the O antigen capsule and biofilm-associated proteins [30-32]. Multicellular phenotypes of *S*. Typhi strains studied in this work were further characterised for the expression of curli fimbriae on Congo Red Agar. Colony morphologies on Congo Red plates were scored according to the basic morphotypes previously detected in *S*. Typhimurium [33]: RDAR (violet colony, expresses curli fimbriae and cellulose), PDAR (pink colony, expresses cellulose), BDAR (brown colony, expresses curli fimbriae) and SAW (no expression of curli fimbriae nor cellulose). However, in our work, we have observed only RDAR (Figure 3A and B) in all 60 strains of *S*. Typhi originated from different countries, years and samples. There was no variation observed in RDAR among *S*. Typhi strains from blood or stool. RDAR morphotype is commonly observed in the other *Salmonella enterica* including *S.* Typhimurium and *S.* Enteritidis [34]. White and Surette [35] reported that all 7 *Salmonella* subspecies studied in their work expressed RDAR morphotype which is a distinct, rough and dry colony morphology formed by the extracellular interaction of thin aggregative fimbriae (Tafi or curli), cellulose, and other polysaccharides. In another study, Romling and co-workers also observed RDAR morphotype in all *Salmonella* serovars tested [36].

Fimbriae or curli plays a vital role in attachment of the bacteria to the surface and gives a signal for initiation of microcolony formation. Many bacterial pathogens use subcellular surface appendages that radiate from the bacterial surface for initial adherence. Typical examples are the bacterial pili (fimbriae) and flagella. In *E. coli*, mutation in type 1 fimbriae had severe defect in initial attachment [37]. *S*. Agona strains with BDAR morphotype was found to be equally tolerant to disinfection treatment as strains with RDAR morphotype in biofilm formation test. Both BDAR and RDAR morphologies were good biofilm producers, however, no statistical difference was found between the two morphotypes [38]. The RDAR morphology appeared to be favourable in long term survival in biofilm in a very dry environment [38]. It has previously been shown that the RDAR morphology is due to the expression of both fimbriae and cellulose contributing to a highly organised structure, and this organised structure is disrupted at the loss of one of these components [34,39,40]. Our hypothesis is that these structures might be of importance for the long term survival of *Salmonella*.

### *S*. Typhi strains demonstrated variations in biofilm formation ability

It has been reported that motility is required for both biofilm formation and pathogenesis [41]. Therefore, we examined the *in-vitro* biofilm-forming ability of the 60 *S.* Typhi strains using crystal violet assay in 96-well microtitre plate. We observed a wide variation in the quantity of biofilm biomass produced amongst the strains. Approximately one third (of the *S*. Typhi strains tested (n =20) were not able to adhere to the plastic wells of the 96-well microtitre plate, indicating inability to form biofilm *in-vitro*; another one third of the strains (n =21) were only able to produce weak biofilm in the *in-vitro* assay; 12 strains (20%) were moderate biofilm producers; and only 7 strains strongly adhered to the inner walls of the plastic wells, representing potentially strong biofilm producers (A_590_ nm > 1.3). Although almost two thirds of the strains tested were non- or weak biofilm producers, these strains may still be important during polymicrobial infections where they can directly be incorporated into an established biofilm or interact with other species providing synergy to the non-biofilm formers. All the 7 strong biofilm formers were highly motile, in which, 5 *S*. Typhi strains originated from Malaysia, 1 from Indonesia and 1 from Chile. Only 1 carrier strain CR0063/07 from Malaysia was recorded to have strong biofilm forming ability and the other 6 *S*. Typhi strains were isolated from blood and environmental samples. Asymptomatic carriers of *S*. Typhi periodically shed large numbers of this bacterial pathogen in their stools (showers of *S*. Typhi). Because of the hallmark showers of *S*. Typhi, carrier identification requires collection and culture of multiple faecal samples over the period of at least 1 year. Due to this difficulty in isolation, we had only 2 human carrier strains among the 60 *S*. Typhi strains studied. In one of the previous studies, *P. aeruginosa* strains with a mutation in type IV fimbriae did not form a densely packed biofilm. The type IV fimbriae appear to play a role in full biofilm formation by the bacteria [42]. These data could suggest that fimbriae are needed during stages of intestinal and gallbladder infection of *S.* Typhi but specific environmental signals such as bile and other factors may play an important role in their regulation and therefore it affects the level of biofilm forming ability.

**Figure 4:**
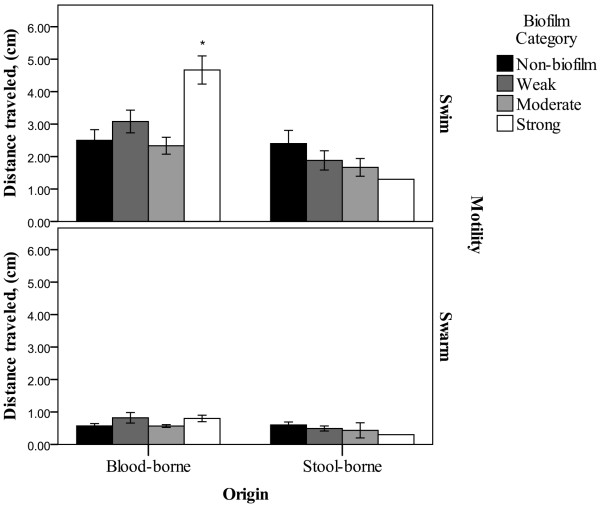
**Blood-borne and stool-borne *Salmonella *Typhi in swim and swarm motility under different biofilm formation category.** Biofilm category is represented by the different colour codes on bar charts. Y-axis indicates the distance traveled (cm) in swarm and swim motility assays. Vertical lines associated with histogram bars represent standard error of the mean. There was a significant difference observed only in swim motility assay under strong biofilm category (* implies *p* < 0.05) for blood-borne strains.

In our work, when we plotted the *in-vitro* biofilm forming ability against motility of the 60 *S*. Typhi strains tested, we did not observe any negative relationship between bacterial biofilm forming ability and motility as reported in other publications [43,44]. However, when we plotted the graph separately for blood-borne (n =24) and stool-borne (n =19) *S*. Typhi strains, a negative trend was observed only in the stool-borne strains, but not in the blood-borne *S.* Typhi strains. In most of the published studies, those non-*Salmonella* bacterial strains were isolated from the environment (e.g. soil, water) [45,46]. In this study, a similar negative trend was observed for environmental strains in which highly motile *S.* Typhi strains were either weak or moderate biofilm formers. The Chilean strains that were studied were from clinical and environmental sources. However, there was no difference observed between the clinical and environmental strains. These clinical strains were from stools and they behave similarly as the environmental strains. But only one Chilean strain from blood was able to express high motility compared to other Chilean strains. The results also indicated that the blood-borne *S*. Typhi strains were able to swim better as compared to those from stool samples (F = 9.026; P = 0.005) (Figure 4).

To explain the negative trend between motility data and biofilm, we found a study conducted by Crawford and co-workers [18]. They demonstrated that mutations in serovar Typhimurium flagellum structural and biosynthesis genes affected binding and biofilm formation on cholesterol. To determine if the physical presence of the flagellar filament or flagellum-mediated motility was required for biofilm formation, mutants that expressed flagella but could not swim were tested. In the tube biofilm assay, a serovar Typhimurium *motA* mutation (which eliminates flagellar motility but not synthesis) did not reduce the levels of biofilm on cholesterol surfaces in the presence or absence of bile compared to the results obtained for the parent strain, suggesting that motility is not critical for development of serovar Typhimurium biofilms on cholesterol coated surfaces. However, in the same study, to examine whether production of the flagellar filament is necessary for biofilm formation on cholesterol, a mutation in the gene at the apex of transcriptional regulation (*flhC*) in serovar Typhimurium was created. The resulting mutant strain did not form a mature biofilm on cholesterol, providing direct evidence of the importance of flagella during biofilm development. Therefore, motility provides various contributions to biofilm formation in members of the Enterobacteriaceae, such as *V. cholerae, E. coli* and *P. aeruginosa,* depending on the environmental conditions, such as binding substrate material, nutrient limitation, temperature, medium flow rate, and other factors.

According to previous studies [24,47,48], a lag period of non-motile behaviour precedes the initiation of swarming motility when bacteria are transferred from a liquid medium to a solid surface. The swarming lag is constant for a particular set of conditions but may be shortened and therefore some strains from blood-borne origin are able to swim well compared to strains from stool origin. Although, the swarming lag is poorly understood, its occurrence indicates that swimming cells must go through a change to become swarming proficient. The difference in the surface swarming ability between both origins was not statistically significant (F = 3.958; P > 0.05). It was obvious from the result that blood-borne *S*. Typhi strains could swarm better than the stool-borne strains (Figure 4). However, we did not observe any difference in the *in-vitro* biofilm forming ability between blood- and stool-borne *S*. Typhi strains (Figure 4).

The differences observed between blood- and stool-borne *S.* Typhi strains in this study suggest that *S.* Typhi strains demonstrated different physiology during the invasive infection stage (blood-borne) and acute infection stage (stool-borne). This observation was indeed intriguing, as *S.* Typhi strains isolated from stool and blood actually represent two different stages in human infection and colonisation niches within the human body. *S.* Typhi is able to invade the intestinal wall and replicate within macrophages and infected phagocytes [49]. The replication of the bacteria within macrophages in the liver and spleen resulted in the release of the pathogen into the bloodstream [29,50]. The pathogen later invades the gallbladder and leads to bacterial shedding in urine and faeces in the chronic carriage of infected individuals [51,52]. It is possible that *S.* Typhi acquires different metabolic activity and phenotypes for colonisation and persistence in these two different niches, liver and spleen, which then disseminate *S.* Typhi into the blood stream and the gallbladder (which *S.* Typhi is then released in the urine and faeces), respectively.

**Figure 5:**
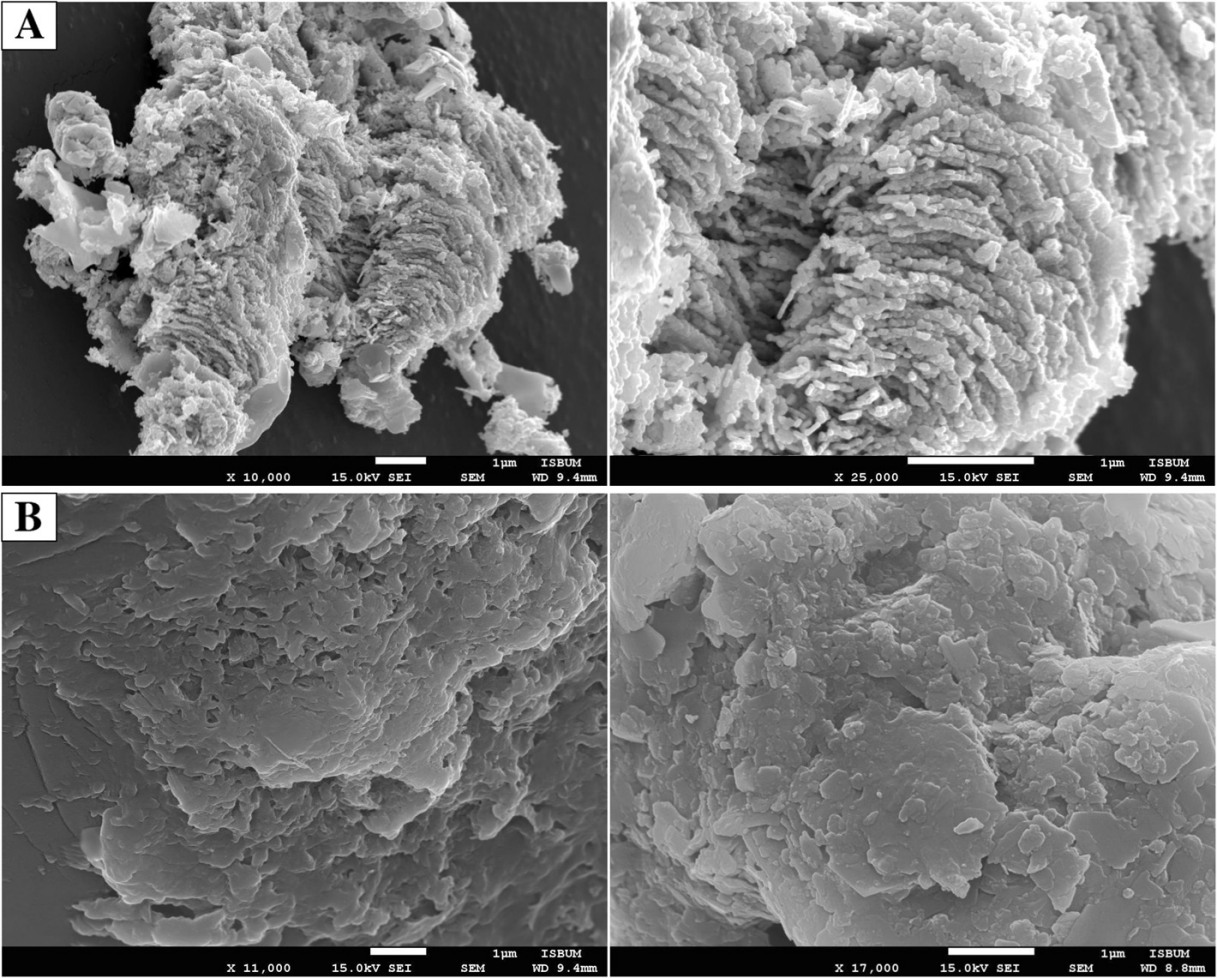
**SEM micrographs of *S*. Typhi on a 96-well polystyrene peg's surface.** Panel **A** shows SEM micrographs of a human carrier strain (CR0063/07), embedded in biofilms established on the polystyrene peg's surface at magnifications of 10,000x and 25,000x. Panel **B** shows SEM micrographs of an outbreak *S*. Typhi strain (BL196/05). Biofilm formation was not detected on the polystyrene peg's surface at magnifications of 11,000x and 17,000x.

In this study, we selected two strains, one of human carrier origin (stool-borne; CR0063/07) while another one of outbreak origin (blood-borne; BL196/05) to compare their biofilm structure and architecture using the scanning electron microscopy (SEM). The bacterial biofilm was allowed to grow and form on the peg lids of a 96-well plate as described previously by Harrison *et al.*[53]. Only the carrier strain (CR0063/07) was capable of forming mature, robust biofilms on the polystyrene peg's surface. Bacterial cells of *S*. Typhi adhered to each other and were encased in an extracellular matrix in the biofilm formed by the carrier strain; whereas there was no biofilm formation in the outbreak strain, extracellular matrix was not observed too (Figure 5). Could this observation suggest a possible persistence strategy of the carrier strain in the human host? More in depth studies are needed to answer this interesting question.

### Conclusion and future directions

In summary, both swimming and swarming motility are conserved among *S*. Typhi strains but there was variation for biofilm forming ability. There was no difference observed in this phenotype for *S*. Typhi strains from diverse background. These findings serve as caveats for future studies to understand the lifestyle and transmission of this pathogen.

## Materials and methods

### Bacterial strains

A total of 60 *S.* Typhi strains previously characterised were studied [54-56]. The strains were isolated from various sources including blood and stool samples from typhoid patients, asymptomatic human carrier and sewage-contaminated river water that were collected from Malaysia, Indonesia, Papua New Guinea and Chile, from 1983 to 2008. The strains were retrieved from - 80°C stock cultures, and reconfirmed as *S*. Typhi using an in-house PCR assay. Serotyping was previously done by the Salmonella Reference Centre at the Institute for Medical Research, Malaysia.

### Swarming and swimming capability

To test on motility, a sterile needle was used to lightly touch an overnight *S.* Typhi culture and spotted gently in the middle of a swarm plate (Nutrient Broth [NB], 0.5% [wt/vol] glucose, 0.5% bacteriological agar) or a swim plate (NB, 0.5% glucose, 0.25% bacteriological agar). The plates were incubated at 37°C for 24 hr. The rates of motility were measured and patterns of swarming and swimming on the agar plate were determined according to Kim and Surette [22]. Rates of the migration of bacteria from the point of inoculation (observed as a turbid zone in centimetres) were measured at 6, 12, 18 and 24 hrs. The results are the means of at least 3 independent experiments.

### Biofilm formation of *S*. Typhi in 96-well microtiter plates

To check for biofilm forming ability, the microtitre plate assay used in this study was adapted from O'Toole and Kolter [57], with some modifications. *S.* Typhi cultures were grown in Luria Bertani (LB) broth until mid-log phase. Each strain was then inoculated into 8 wells of 96-well microtitre plate and incubated for 48 hrs. After incubation, unbound cells were removed by inversion of microtiter plate, followed by vigorous tapping on absorbent paper. Subsequently, adhered cells were heat fixed in an oven for 30 min at 80°C. Adhered cells were stained by addition of 220 μl of crystal violet (0.5%) for 1 min. The stain was removed by thorough washing with distilled water. In order to quantify adhered cells, 220 μl of decolouring solution (ethanol/acetone, 80:20%) was added to each well for 15 min. The absorption of the eluted stain was measured at 590 nm wavelength. Based on the O.D_590 nm_, strains were classified into the following categories: no biofilm producer, weak, moderate or strong biofilm producer, as previously described by Stepanović and Ceri *et al.*[58,59]. Briefly, the cut-off O.D. (O.D.c) was defined as three standard deviations above the mean O.D. of the negative control. Strains were classified as follows: O.D. ≤ O.D.c = no biofilm producer, O.D.c < O.D. ≤ (2 × O.D.c) = weak biofilm producer, (2 × O.D.c) < O.D. ≤ (4 × O.D.c) = moderate biofilm producer and (4 × O.D.c) < O.D. = strong biofilm producer. The negative control wells contained nutrient broth only. Negative control wells remained negative. The negative control has been deducted from the OD readings. All tests were performed at least three independent times to ensure reproducibility. Replicates for each test were conducted to check for repeatability.

### Curli and cellulose detection in *S.* Typhi

To substantiate the findings of multicellular phenotypes, colonial morphology of *S*. Typhi bacteria was studied on LB_w_ [1.0 g Tryptone, 1% agar, distilled water to 100 mL without salt supplement containing Congo red (40 μgml^-1^)/ Coomassie brilliant blue (20 μgml^-1^)] (Sigma Chemicals, St Louis, MO, USA) was used to determine colony morphology and colour [36]. *S*. Typhimurium was used as a control on Congo Red Agar to test for RDAR morphotype.

### Primer design and PCR assay

Flagella-associated genes, *fliC* and *flgK* were selected to confirm the motility in all 60 *S*. Typhi strains. Oligonucleotide primers specific for each target gene were selected using PrimerSelect (DNASTAR; Lasergene, Madison, WI). Selected primer pairs were then tested using *in silico* with the PCR amplification program (http://insilico.ehu.es/.25). The sequences of the selected primer used were *fliC* (5'-AAT CAA CAA CAA CCT GCA GCG- 3') and (5'-GCA TAG CCA CCA TCA ATA ACC-3'); *flg*K (5'- CAA CAA TTA CGC GAA GCA GA -3') and (3'- TAT AAT CCG TCG CCT GAA CC -5') with amplicon size 704 bp and 584 bp, respectively. The PCR amplifications were carried out in a Master cycler (Eppendorf, USA). The PCR mixture in a total volume of 25 μl contained 50 ng of genomic DNA template, 1X PCR buffer, 2 mM MgCl_2_, 200 μM of each dNTP, 0.3 μM of each primer, and 0.5U of *Taq* DNA polymerase (Promega, USA). The cycling con ditions were set at 95°C for 5 minutes (1 cycle), 95°C for 30 s, 55°C for 30 s, 72°C for 1 minute (30 cycles), and 72°C for 8 minutes (1 cycle). The products were then analysed on 1.5% (w/v) agarose gel and run at 100 V for 25 minutes and stained in GelRed. Gel images were captured and analysed using Gel Doc XR (Bio Rad, USA). Selected amplified DNA products were verified by DNA sequencing.

### Scanning electron microscopy analysis of carrier and outbreak *S.* Typhi

To determine the architecture of biofilm producers, the Scanning Electron Microscopy (SEM) was conducted on an outbreak and carrier *S*. Typhi strains, both of which demonstrated significant biofilm formation. Bacterial biofilms were grown on polystyrene pegs (Nunc-TSP; Nunc) for 48 hr [60]. Briefly, following incubation, the pegs were rinsed with 1X PBS and removed using sterile needle-tipped pliers. Each peg was then fixed with 2% (w/v) glutaraldehyde, 2% (w/v) paraformaldehyde, 0.15 M sodium cacodylate, 0.15% (w/v) alcian blue for 3 hrs at room temperature. Pegs were then rinsed three times with 0.15 M sodium cacodylate buffer, immersed in 1% (v/v) osmium tetroxide in sodium cacodylate and incubated for 1 hr at room temperature. Pegs were then rinsed three times with distilled water followed by a stepwise dehydration with graded ethanol-water mixtures. Samples were then treated with hexamethyldisilizane for 5 min prior to critical point drying. Next day, samples were sputter coated with gold and viewed by SEM. SEM experiments were carried out in duplicate for each strain tested, and representative images of biofilms were selected.

### Statistical analysis

Statistical analyses were performed using t-test for independent samples. All the experiments were repeated at least three times. The level of significance was set at *P* < 0.05. Microsoft Excel and SPSS 18 were used for analysis.

## Competing interests

The author’s declare that they have no competing interests.

## Authors’ contributions

KK performed the motility assays, biofilm assays and SEM assays on *Salmonella* Typhi, analysed the data, performed statistical analyses and drafted the manuscript. LCC supervised all experimental work, analysed the data, and drafted the manuscript. KLT conceived the study, supervised all experimental work, analysed the data and drafted the manuscript. All authors read and approved the final manuscript.
